# Developing and validating key performance indicators for breast, cervical, and colorectal cancer screening programs: a literature review and Delphi survey

**DOI:** 10.3389/fpubh.2025.1491226

**Published:** 2025-04-03

**Authors:** Arezoo Jabbari, Zhila Najafpour, Sima Ourang, Maria Cheraghi

**Affiliations:** ^1^Department of Health Care Management, School of Public Health, Ahvaz Jundishapur University of Medical Sciences, Ahvaz, Iran; ^2^Department of Health Care Management, School of Public Health, Social Determinants of Health Research Center, Ahvaz Jundishapur University of Medical Sciences, Ahvaz, Iran; ^3^Department of Non-Communicable Diseases, Deputy of Health, Ahvaz Jundishapur University of Medical Sciences, Ahvaz, Iran; ^4^Department of Public Health, School of Public Health, Ahvaz Jundishapur University of Medical Sciences, Ahvaz, Iran

**Keywords:** cancer screening program, key performance indicator, systematic review, content validation, Delphi

## Abstract

**Introduction:**

Early detection of cancer significantly impacts disease management and contributes to a reduction in cancer mortality rates. This study aims to identify, extract, systematize, and validate a set of indicators for breast, cervical, and colorectal cancer screening programs that are applicable and easily understood within any healthcare system.

**Methods:**

This study is conducted in two phases: a literature review and an expert panel evaluation. In the first phase, electronic databases—PubMed, Web of Science, and Scopus—were searched for articles published from January 2000 to November 2023. Two reviewers critically appraised the articles based on predefined inclusion and exclusion criteria. Indicators were extracted from the selected articles through content analysis. In the second phase, the extracted indicators were reviewed by ten experts. Consensus on the indicators was achieved through two consecutive rounds of review.

**Results:**

The final list comprises 30 indicators categorized into three dimensions: two for input, sixteen for process, and twelve for outcome. The overall content validity index (CVI) and content validity ratio (CVR) determined using the expert panel agreement approach, were high (≥ 0.9). The input dimension includes two indicators: Adequacy and Availability of Human Resources, and Percentage of Health Centers Providing Cancer Screening Services. The process dimension comprises 16 indicators, including Timely Diagnostic Evaluation of Abnormal Screenings, Rescreening, Recall Rate, Percentage of Smears per 1,000 Women Aged 20–29 per Year, Public Education, Data Availability, Referral Rates (to GP and Surgeon), Drop Rate During Referral, Biopsy Rate, Diagnostic and Therapeutic Endoscopy Rate, Proportion of Colonoscopies, Total and Partial Mastectomy Rates, Tumor Diameter, and Tumor Grading. Finally, the outcome dimension features 12 indicators: Screening Coverage, All-Cause Mortality Rate, Cause-Specific Mortality Rate, Invasive Cancer Detection Rate, Interval Cancer Rate, Ductal Carcinoma *in Situ* (DCIS) Rate, Cancer Detection Rate, Polyp Detection Rate, Fecal Occult Blood Test (FOBt) Positivity Rate, Adenoma Detection Rate, Positive Predictive Value for Cancer Detection (PPV), and Episode Sensitivity.

**Conclusion:**

This study identified a robust set of 30 key performance indicators (KPIs) for breast, cervical, and colorectal cancer screening programs, with a high overall content validity index demonstrating strong expert consensus on their relevance and importance.

## Introduction

Cancer disease is one of the biggest public health challenges that increases rates of mortality and morbidity, with an estimated 19.3 million new cases and 10 million deaths in the world in 2020 (1). Estimates in Iran show since 2016, the trend of cancers has been increasing, and this growth is expected to continue until 2040, when the number of cancers will more than double compared to 2020 (2). Cancer prevention and control is one of the most important priorities in any health system around the world. Based on the evidence, early detection of cancer has a considerable impact on managing the process of disease management and consequently reducing the cancer incidence and mortality rate (3).

Cancer screening accelerates early detection before a person develops symptoms and increases the opportunity for treatment. Cancer screening is the use of a test among the target population with a higher probability of cancer (4). Not all of screening programs have evidence of effectiveness; it is a trade-off between cost, burden, and risk of cancer. In other words, cancer screening is launched when the risk of cancer is high to justify the investment of resources. Additionally, it should have supportive evidence from valid studies (5). The implementation of screening programs for cancers needs strong governance, a comprehensive program for cancer management, allocation of appropriate financial, human, and technical resources, information system for monitoring and evaluation of the program.

Screening programs for cancers can be conducted in different contexts, including doctors’ offices, community settings, and population-based national programs. Based on the literature, many of these plans are not successful due to *inadequate* financial resources, a lack of a scaling-up strategy, political willingness, and stakeholder engagement (6). Any cancer screening program aims to reduce mortality and morbidity in a population through the early detection and treatment of cancer (7).

A screening program is not just a single test but a pathway. It starts with identifying the people who are eligible for screening, referred to as the target population. The program pathway includes diagnosis and treatment. Screening programs for several cancers like breast, cervical, and colorectal, have been launched in various developing countries following the experiences of developed countries (6). Iran’s National Cancer Control Program (IRCCP) was developed comprehensively in 2013 with cross-sectoral cooperation and stakeholder participation (2). The IRCCP as a strategic program is designed to meet the needs of the population by preventing, diagnosing, and treating cancer as well as provide care for patients (8). This program focuses on early detection of breast, cervical, and colorectal cancers (2).

Scientific literature from various countries indicates that national cancer screening programs have often been less effective due to several factors. In South Korea, reports highlight low positive predictive values (PPV) and sensitivity rates, raising concerns about the program’s reliability and its actual impact on reducing cancer-related mortality rates (9). Similarly, in Tanzania, patients completed about half the test referrals for further diagnostic investigations, highlights significant issues related to follow-up and continuity of care (5). Reports from China identify low participation rates as a primary barrier to the success of its national cancer screening initiatives (10).

The final step in the pathway is the evaluation of the cancer screening program (7). Based on our assessments, several reviews have addressed indicators for cancer screening programs evaluation. For instance, Mema et al. (11) concentrated solely on indicators of participation within cancer screening programs. Csanádi et al. (12) examined indicators in organized screening programs without exploring the practical and feasibility issues of data collection. Wang et al. (13) developed indicators specifically to evaluate access to screening services, and Ding and Mosquer focused on performance indicators of colorectal cancer (CRC) screening programs (14, 15). Bruni et al. (16) emphasized screening coverage as a core indicator in cervical cancer programs, and Carballo et al. (17) evaluated quality indicators within breast cancer clinical pathways. Additionally, Selby et al., identify guidelines from screening programs containing quality metrics for tests used in breast, cervical, colorectal, and lung cancer. They also considered the publication time frame of studies conducted between 2010 and 2020 (18). In our review, we take a more comprehensive approach to identify cancer screening indicators across three major cancers—colorectal, cervical, and breast—and validate the extracted indicators for robust application in both practice and policy.

Key performance indicators (KPIs) are as essential tools for assessing the effectiveness and efficiency of cancer screening programs. They provide measurable benchmarks that facilitate the monitoring, refinement, and enhancement of program outcomes. By identifying KPIs, this research offers policymakers data-driven insights to guide resource allocation, set achievable performance targets, and strengthen program accountability. This study aims to identify, extract, systematize, and validate a set of indicators for Breast, Cervical, and Colorectal cancer screening programs that are applicable and easily understood within any healthcare system.

## Materials and methods

This study was conducted as a systematic review to extract key performance indicators for breast, cervical, and colorectal cancer screening programs and a Delphi study including a panel of 10 experts to validate the indicators.

### Systematic review phase: defining key performance indicators (KPI) of cancer screening programs

In the review part, articles published and indexed the electronic databases of PubMed, Web of science, and Scopus were searched from Jan 2000 to Nov. 2023. Our search was performed with a combination of keywords “Cancer,” “Cancer Screening,” “Program Assessment, “Cancer Early Detection,” “screening program” (see the complete search strategy in the Appendix 1).

### Inclusion and exclusion criteria

Observational and comparative studies, prospective, retrospective, global, and national reports that have discussed indicators of cancer screening programs were considered eligible for inclusion. We included all of the studies that provided indicators to assess breast, colon, and cervical cancer screening programs. The criteria for exclusion included studies that did not clearly define or report indicators for monitoring the performance of the selected cancer screening programs, as well as letters and conference abstracts. Furthermore, articles published in languages other than English or Persian were excluded.

### Study selection and data extraction

All of the databases were independently searched by two researchers (Z.N. and A.J.), and the search results were entered into EndNote software. Following the removal of duplicate records (*n* = 20), the titles and abstracts of the remaining articles were screened. Irrelevant articles were excluded (*n* = 2,497), and the full texts of the relevant articles were then assessed for eligibility. Data extraction was carried out independently by the two researchers (Z.N. and A.J.). The extraction process was guided by predefined criteria, including author names, publication year, country and study location, study type, methods of data collection and analysis, and outcome indicators. Any disagreements during the selection, qualitative assessment, or data extraction stages were resolved through discussion to reach consensus.

#### Quality assessment

The quality of the included studies was assessed using the Joanna Briggs Institute (JBI) critical appraisal tools (19) relevant to each study’s methodology. Studies were categorized into three quality levels: low, medium, and high. This process was conducted independently by two researchers (Z.N. and A.J.). Discrepancies were discussed until consensus was reached, with a third researcher involved, if necessary, who also revised those studies considered to have low or medium methodological quality (see Appendix 2).

### Delphi phase: determining content validity of KPI for cancer screening programs

Key performance indicators (KPIs) were initially identified through a comprehensive literature review conducted in the previous phase. The identified indicators were subsequently validated by a panel of ten experts selected based on specific inclusion criteria: a background in public health or healthcare management, and a minimum of 4 years of relevant experience. The panel comprised two managers from the public health deputy, six professors specializing in relevant disciplines including one of whom was the head of the cancer registry system and a specialist in cancer diseases, three specialists in healthcare management and one expert in health policymaking (two with executive backgrounds in the health system), and two researchers with professional experience in this area. Among the participants, two were physicians, seven held Ph.D. degrees, and one had a master’s degree. Data collection occurred over a one-month period, from May to June 2024.

To facilitate better comparisons, we decided to use the Donabedian Structure-Process-Outcome (SPO) framework. According to the Donabedian model, ‘structure’ refers to the environment in which healthcare is delivered, including aspects such as facilities, equipment, and the numbers and qualifications of related human resources for health (HRH). ‘Process’ encompasses the actions involved in providing and receiving care, such as communication between patients and healthcare providers, as well as information sharing. ‘Outcome’ represents the results of the provided healthcare, including factors like patient health status, satisfaction, and costs. The quality of healthcare is evaluated by examining these three categories and their interactions (20, 21). The first draft of the checklist comprised 33 indicators, including two for inputs, sixteen for processes, and fourteen for outcomes (see Appendix 3).

The first draft of the checklist was prepared by the research team in two consecutive meetings and was then finalized in two Delphi rounds. In the first round, we asked the experts to assess each indicator based on four criteria: necessity, clarity, simplicity, and relevance. Additionally, we asked the experts to assess the definitions of the indicators through an open-ended question. After receiving the completed checklists from the experts, one researcher entered the raw data into Excel software, where it was analyzed using descriptive statistics. The aggregated results for each indicator were sent to the participants in the second round to give them an opportunity to refine their opinions. After the final assessment, the indicators were confirmed by the participants.

#### Content validity ratio (CVR)

The Content Validity Ratio for each indicator was calculated using Lawshe’s formula (10). Experts were asked to evaluate each indicator based on the following categories: (1) Necessary, (2) Useful but Not Necessary, and (3) Not Necessary. The responses for each item were then calculated using the following formula:

$CVR=\frac{\left(N_{e}-N/2\right)}{\left(N/2\right)}$

In this formula, N_e_ represents the number of specialists who selected the option deemed ‘necessary’ (1. Necessary, 2. Useful but Not Necessary, and 3. Not Necessary), while N denotes the total number of assessors. A CVR value greater than 0.62 is considered acceptable based on the number of experts involved in the evaluation (22).

#### Content validity index (CVI)

To calculate the content validity index (CVI), experts were asked to assess the clarity, simplicity, and relevance of each indicator on a four-point Likert scale, ranging from “not relevant/unclear” to “completely relevant/clear. A Content Validity Index (CVI) score above 0.79 is considered appropriate; scores between 0.70 and 0.79 are deemed questionable and require revision, while scores below 0.70 are considered unacceptable and should be removed (23).

The responses for each item were then calculated using the following formula:

The CVI is calculated using the following formula:

CVI=Number of rates givingarating of'3'or'4'Total number of assessors

To minimize bias, two researchers independently evaluated the experts’ feedback on the open-ended questions regarding the indicators.

## Results

### Systematic review phase: defining key performance indicators (KPI) of cancer screening programs

**Figure 1:**
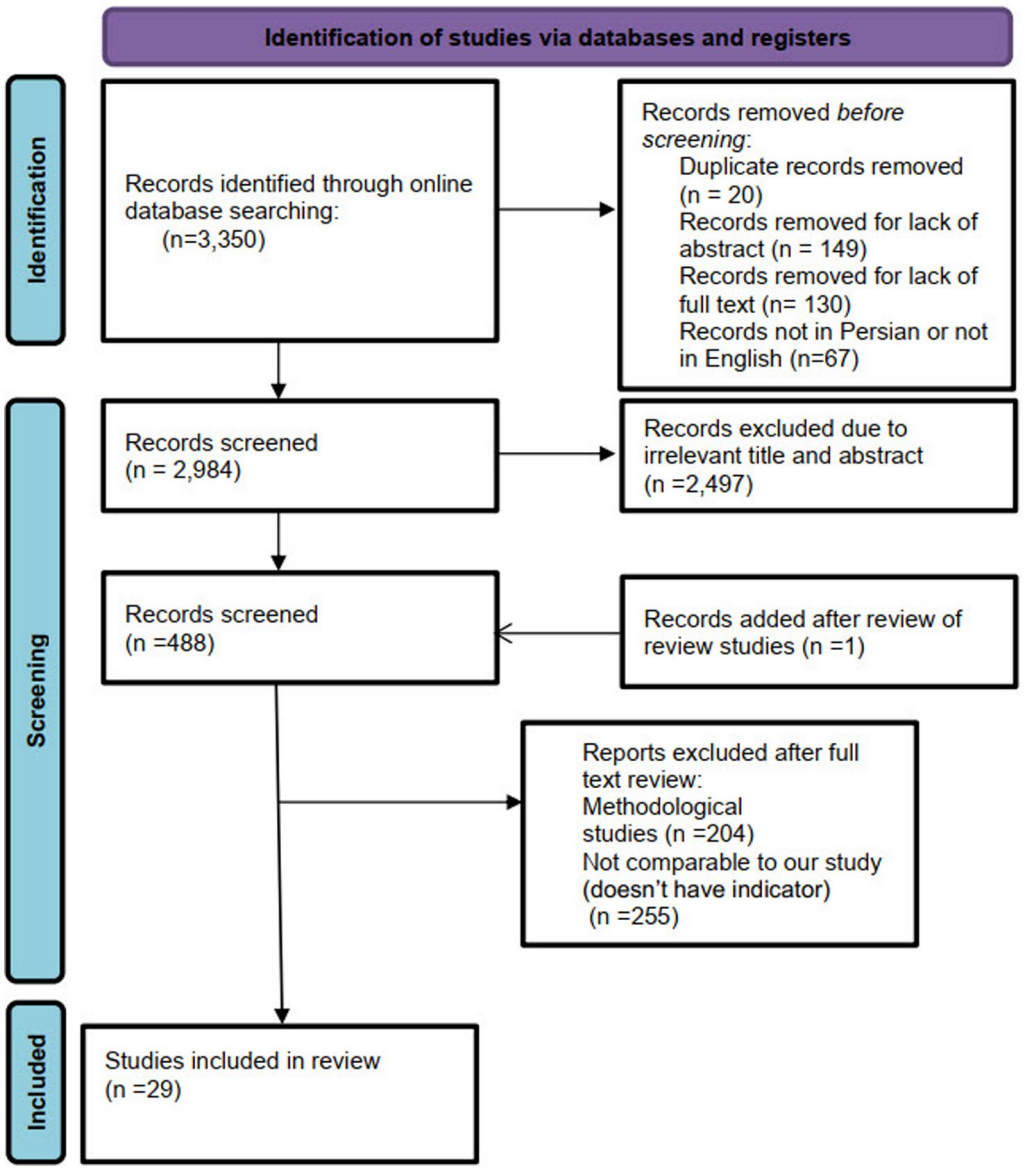
PRISMA flowchart.

A total of 3,350 citations were obtained from the selected databases. After screening the titles and abstracts, 2,497 citations were excluded. A full-text review was conducted for 489 citations, resulting in the inclusion of 29 studies published between 2000 and 2023 that met our eligibility criteria. The PRISMA flowchart detailing the selection process is presented in Figure 1.

The included studies were mainly conducted in 16 different countries consisting Iran (*n* = 5) (2, 24–27), United states of America (USA) (*n* = 4) (28–30), the Netherlands (*n* = 3) (31–33), Canada (*n* = 3) (34–36), Australia (*n* = 2) (37, 38), the United kingdom (UK) (*n* = 2) (39, 40), Norway (*n* = 1) (41), Saudi Arabia (*n* = 2) (42, 43), Serbia (*n* = 1) (44), the Czech Republic (*n* = 1) (45), Portugal (*n* = 1) (46), Hungary (*n* = 1) (12), Colombia (*n* = 1) (47), Brazil (*n* = 1) (48), and Korea (*n* = 1) (49). The majority of the included studies were cross-sectional (73.3%) (see more details in Appendix 4).

Firstly, a total of 325 indicators were extracted from the included studies. Following a thorough assessment, 245 indicators were removed due to redundancy, irrelevance, or repetition. Ultimately, 32 indicators were retained for further analysis (See Appendix 5). The remaining indicators were categorized according to the Donabedian model into three main categories: input, process, and outcome (see Table 1).

**Table 1:** Key performance indicators for cancer screening programs.

Type	Indicator	Screening measure	References
Input	Human resource for health	Sufficient and available human resources	(27)
		Percentage of health centers in which providing cancer screening services	(2)
Process	Timely diagnostic evaluation of abnormal screens	Percentage of abnormal screening results with time from screening test result to final diagnosis >60 days	(30)
	Rescreening	The proportion of women aged 30–69 screened in a given year whose screening outcome was a recommendation to return for screening in 2 years and who returned for a screen within 27 months.	(13, 39, 40)
	Recall rate	Numerator: n° of women undergoing further assessment for medical reasons based on a positive screening examination (either on the same day as screening or on recall) Denominator: n° of women screened	(33, 34, 39, 43, 44, 47–49)
		Percentage of smears per 1,000 women aged 20–29, per year	(35)
	Public education	Percentage of campaigns held to inform and educate the public about cancer prevention	(2)
	data availability	Numerator: No. of women for whom data were available Denominator: n° of women screened	(28)
	Referral rate	Numerator: No. of Women referred to GP Denominator: n° of women screened	(2, 28, 33, 34)
		Numerator: No. of Women referred to surgeon Denominator: No. of Women referred to GP	
	Drop rate of people during referral from GP to surgeon	Numerator: No. of Women referred to surgeon Denominator: No. of referred from GP to surgeon	(28)
	Biopsy rate	Numerator: No. of biopsies Denominator: No. of referred women to biopsy	(34–37, 44, 45, 47–50)
	Diagnostic/therapeutic endoscopy rate	Numerator: No. of with diagnostic or therapeutic endoscopy Denominator: No. of tested population	(25, 29)
	Proportion of colonoscopies	Numerator: No. of colonoscopies Denominator: No. of people referred for colonoscopy	(25, 29)
	Total Mastectomy rate	Numerator: n° of women with Total mastectomy Denominator: n° of women screened	(34)
	Partial Mastectomy rate	Numerator: n° of women with Partial mastectomy Denominator: n° of women screened	–
	Tumor diameters (*n*)	n° of % < 10 mm n° of % < 15 mm n° of % > 20 mm	(48)
	Tumor graded (*n*)	n° of % grade I n° of % grade II n° of % grade III	(48)
Outcome	Screening coverage	Numerator: n° of women screened Denominator: n° of eligible (or target) women within a given period	(2, 13, 26, 28, 29, 33, 34, 46, 49)
	Mortality (all cause)	Numerator: n° of 30-day all-cause mortality Denominator: n° of tested population	(26, 27, 29, 39, 41, 50)
	cause-specific mortality	Numerator: n° of deaths from the given cancer in a population	–
	Invasive cancer detection rate	Numerator: n° invasive screen-detected cancers Denominator: n° of women screened	(33, 34, 36, 39, 40, 43, 44, 47–49, 51)
	Interval cancer rate	Numerator: n° of interval cancers Denominator: n° of screened negative women at the last screening round	(28, 33, 34, 36, 39, 42, 43, 49)
	ductal carcinoma *in situ* (DCIS)	Numerator: n° of DCIS cancers Denominator: n° of cancer detected womens	(33, 34, 36, 39, 44)
	Cancer detection rate	Numerator: n° of all malignant cancers detected every 1,000 screened women Denominator: n° of women screened	(29, 34, 36, 41, 43, 44, 47, 49, 50)
	Polyp detection rate	Numerator: n° of people with polyps Denominator: n° of tested population	(25, 29)
	FOBt positivity rate	Numerator: n° of people with positive FOBt Denominator: n° of tested population	(29)
	Adenoma detection rate	Numerator: n° of people with adenomas Denominator: n° of tested population	(25, 29)
	Positive predictive value for cancer detection(PPV)	Numerator: the ratio of lesions that are truly positive Denominator: those with positive test	(29, 36, 49)
	Episode sensitivity	Numerator: n° of screen-detected cancers Denominator: n° of all cancers detected	(34, 36, 39, 49)

### Quality assessment of articles

Qualitative evaluation of the included articles was conducted using the Joanna Briggs Institute (JBI) criteria for 29 studies. The quality ratings were categorized as follows: a score higher than 70% was considered high quality, a score between 50 and 70% was deemed medium quality, and a score below 50% was classified as low quality (50). Out of the 29 studies assessed, 22 were rated as high quality, while 7 were rated as medium quality (for further details, please refer to Appendix 2). This assessment underscores the robustness of the evidence base used in the study.

### Delphi phase: determining content validity of KPI for cancer screening programs

The content validity index (CVI) and content validity ratio (CVR) were assessed using an expert panel agreement approach, yielding high scores overall (> 0.9). As illustrated in Table 2, the CVI and CVR for each indicator reflect a robust validation process. Out of the 32 indicators identified during the review phase, 28 were retained, while four were modified. The indicators removed from consideration included participation rate and follow-up of positive screened women (process dimension), as well as false positive rate at screening and invitation coverage (outcome dimensions) because of overlapping with other indicators. In response to expert feedback, two new indicators were proposed: cause-specific mortality and partial mastectomy rate. Furthermore, several indicators underwent revisions. Each indicator’s definition was extracted by existing studies and finalized through expert consensus. The final list comprises 30 indicators categorized into three dimensions: two for input, sixteen for process, and twelve for outcome. The details of these indicators are presented below, organized by the three dimensions:

**Table 2:** Content validity index (CVI) and content validity ratio (CVR) results for key performance indicators.

Indicator name	CVI	CVR
Adequacy and Availability of Human Resources	1	1
Percentage of Health Centers Providing Cancer Screening Services	0.97	0.8
Timely Diagnostic Evaluation of Abnormal Screenings	0.97	1
Rescreening	0.9	1
Recall rate	0.90	0.80
percentage of smears per 1,000 women	0.97	1
Public education	0.97	1
data availability	0.90	0.80
Referral rate (to GP)	0.87	1
Referral rate (to surgeon)	0.87	1
Drop rate during referral	0.9	0.80
Biopsy rate	0.87	0.8
Diagnostic/therapeutic endoscopy rate	1	1
Proportion of colonoscopies	0.97	1
Mastectomy rate	0.9	1
partial mastectomy rate	0.9	1
Tumor diameter	0.97	1
Tumor grading	0.93	1
Screening coverage	0.97	1
All-cause mortality rate	0.97	0.8
Invasive cancer detection rate	0.9	1
Interval cancer rate	1	1
ductal carcinoma in situ (DCIS)	0.87	0.80
Cancer detection rate	0.93	0.8
Polyp detection rate	0.93	1
FOBt positivity rate	0.97	1
Adenoma detection rate	0.87	1
Positive predictive value for cancer detection (PPV)	0.87	0.8
Episode sensitivity	0.93	0.8
Mean	0.93	0.92

The input dimension includes two key indicators:

1. Adequacy and Availability of Human Resources: sufficient and available human resources.
2. Percentage of Health Centers Providing Cancer Screening Services: number of health centers and bases where cancer screening is given.

These indicators are crucial for evaluating the foundational resources necessary for effective cancer screening programs.

The process dimension consists of 16 indicators, which are essential for monitoring the effectiveness and efficiency of cancer screening processes. These indicators include:

Applicable for all cancer screenings:

1. Timely Diagnostic Evaluation of Abnormal Screenings: percentage of abnormal screening results with time from screening test result to final diagnosis >60 days.
2. Rescreening: the proportion of people aged 30–69 screened in a given year whose screening outcome was a recommendation to return for screening in 2 years and who returned for a screen within 27 months.
3. Recall rate: number of people undergoing further assessment for medical reasons based on a positive screening examination (either on the same day as screening or on recall) / of women screened.
4. Public education: campaigns held to inform and educate the public about cancer prevention
5. Data availability: measures the accessibility and completeness of data related to cancer screening outcomes.
6. Referral rate (to GP): number of people referred to GP for further examination.
7. Referral rate (to surgeon): number of people referred to surgeon for further examination.
8. Drop rate during referral: monitors the percentage of individuals who do not complete the referral process from general practitioners to surgeon.
9. Tumor diameter: assesses the size of tumors at diagnosis, which can impact treatment decisions and outcomes (n° of % < 10 mm, n° of % < 15 mm, n° of % > 20 mm).
10. Tumor grading: evaluates the classification of tumors based on their characteristics, which can influence prognosis and treatment options (n° of % grade I, n° of % grade II, n° of % grade III).
11. Biopsy rate: number of open and core biopsies per 1,000 screens.

Location-specific cancers:

Focus on breast cancer:

12. Total mastectomy rate: tracks the overall rate of total mastectomies performed.
13. Partial mastectomy rate: measures the frequency of partial mastectomies conducted.

Focus on colorectal cancer:

14. Diagnostic and therapeutic endoscopy rate: evaluates the frequency of endoscopic procedures conducted for diagnosis or treatment.
15. Proportion of colonoscopies: indicates the percentage of eligible individuals receiving colonoscopy screenings.

Focus on cervical cancer:

16. Percentage of smears per 1,000 women: evaluates the volume of cervical smears performed in Women.

The outcome dimension includes 12 indicators that evaluate the effectiveness and impact of cancer screening programs. These indicators are:

Applicable for all cancer screenings:

1. Screening coverage: measures the percentage of the eligible (or target) population that has received screening services.
2. All-cause mortality rate: is the rate of All-Cause Mortality within 30-days per 100,000 estimated resident population.
3. Cause-specific mortality rate: is the rate of Mortality caused by breast, colon, cervical cancers per 100,000 estimated resident population in a 12-month period by 5-year age groups.
4. Invasive cancer detection rate: Number of invasive cancers detected per 1,000 screens.
5. Interval cancer rate: is the rate of invasive cancers detected during an interval between two screening rounds per 10,000 women-years.
6. Overall cancer detection rate: Number of all malignant cancers detected every 1,000 screened.
7. Positive Predictive Value for Cancer Detection (PPV): proportion of suspicious cases confirmed as cancer after diagnostic evaluation.
8. Episode sensitivity: proportion of cancer cases (invasive or DCIS) that were correctly identified as having cancer during the screening episode.

Location-specific cancers:

Focus on breast cancer:

9. Ductal carcinoma *in Situ* (DCIS) rate: Number of ductal carcinoma *in situ* (DCIS) cancers detected per 1,000 screens.

Focus on colorectal cancer:

10. Fecal Occult Blood Test (FOBt) Positivity Rate: indicates the percentage of positive FOBt results, which can lead to further diagnostic procedures.
11. Adenoma Detection Rate: evaluates the rate at which adenomas are identified during screenings, important for colorectal cancer risk assessment.
12. Polyp Detection Rate: measures the frequency of polyps detected during screening.

## Discussion

Cancer screening programs are beneficial for the early diagnosis of diseases and the provision of timely medical services. The performance of these screening programs should be monitored using indicators to assess the quality of the screening programs. This study is conducted to identify a set of key performance indicators of cancer screening programs. The overall content validity index (CVI) and content validity ratio (CVR) determined using the expert panel agreement approach, were high (≥ 0.9). The final list comprises 30 indicators categorized into three dimensions: two for input, sixteen for process, and twelve for outcome. The common outcome indicators among breast, cervical and colorectal cancers are: screening coverage, invitation coverage, mortality (all cause), cause-specific mortality, invasive cancer detection rate, interval cancer rate, ductal carcinoma *in situ* (DCIS), cancer detection rate, episode sensitivity and positive predictive value for cancer detection (PPV). Several indicators, including polyp detection rate, diagnostic/therapeutic endoscopy rate, proportion of colonoscopies, FOBT positivity rate, and adenoma detection rate, are specific to colorectal cancer. The mastectomy and ductal carcinoma in situ (DCIS) rates are specific indicators for breast cancer, while the percentage of smears per 1,000 women serves as a specific indicator for cervical cancer.

Recall and cancer detection rates are important as performance indicators for evaluating cancer screening programs. The point is with any screening test, it is important to minimize overutilization of unnecessary downstream diagnostic procedures. A high recall rate leads to psychological distress and suggests inefficient use of screening resources, resulting in unnecessary and costly follow-up investigations (42). A 2017 study by Grabler et al. (51) suggested that a recall range from 12 to 14% is optimal for cancer detection. In contrast, other study considered 3.1% for the optimum incident screening recall rate (52). Nevertheless, international comparisons are difficult because of differences in screening requirements. Then, there is no consensus about what the optimal recall call rate should be. Further research is needed to establish a universally accepted optimal recall rate for cancer screening.

The indicators of referral rate and drop rate during the referral process from general practitioners (GPs) to surgeons are crucial for effectively monitoring suspicious cases. A study by Sediqi et al. conducted in Iran found that the referrals drop rate from health centers to GPs was 8%, while the drop-out rate from GPs to surgeons was 75%. This significant decline in individuals suspected of having a breast mass at various stages of the referral process—particularly from GPs to surgeons—demonstrates that the healthcare system has not succeeded in providing comprehensive follow-up for these patients (24).

A comprehensive screening program should encompass the entire target population and provide screening services to all eligible individuals. The screening coverage index is crucial for evaluating this aspect. Various studies have highlighted the significance of this index in assessing the effectiveness of screening programs. Research has emphasized the importance of targeted systems for inviting individuals to participate in screening programs (12, 32, 47). For instance, a specific indicator for cervical cancer is the percentage of smears performed per 1,000 women aged 20–29, which reflects the coverage of the screening program. There is a notable disparity in screening rates, with coverage ranging from 84% in high-income countries to 9% in low-income countries (16).

The interval between screenings is another indicator affecting cancer detection rate. Some studies recommend either shortening interval between screenings or extending the age ranges for screening. Extending intervals may allow tumors to progress to a stage where symptoms become apparent thereby diminishing the benefits of early detection. In the other hand, shorter intervals may help avoid unnecessary invasive tests for patients (38). A high re-screening rate is essential for increasing the likelihood of early cancer detection and maintaining overall participation in screening programs (37). For example, a study conducted by Evina Bolo in 2023, which evaluated the re-screening rate among women, found that only 34% of the target population participated in re-screening. The study identified key barriers to re-screening and follow-up as lack of information, forgetfulness, and the perception of being healthy (53).

The ultimate objective of any cancer screening program is to reduce cancer-associated mortality. In line with this goal, two key indicators are assessed: all-cause mortality and cause-specific mortality. Evaluating mortality rates among both screened and unscreened populations is crucial for understanding the impact of screening. There are potential biases associated with using either all-cause mortality rates or cancer-specific mortality rates to evaluate screening programs. Based on a systematic review, cancer screening trials are in theory able to demonstrate a significant reduction in all-cause mortality due to screening (54). Conversely, Stang et al. (55) report cancer screening may have a minimal effect on all-cause mortality. Therefore, the effectiveness of cancer screening in reducing all-cause mortality remains a subject of ongoing debate.

The positive predictive value for cancer detection (PPV) is defined as the proportion of cases with suspicious findings that are diagnosed with cancer either invasive or ductal carcinoma *in situ* (DCIS), following a diagnostic work-up. PPV serves as an indicator of the predictive validity of the screening process. Factors that influence cancer detection rate and abnormal call rate must also be considered when evaluating a program’s PPV. Typically, PPV improves with subsequent screenings, as the initial screen establishes a normal baseline. Therefore, PPV is generally lower for initial screenings compared to follow-up screenings.

### Limitation

This study has some limitations. First, our review focused exclusively on articles published in English and Persian. Then we might have failed to identify relevant data published in local languages. Second,

All experts work within the healthcare system in Iran, which may influence the applicability of the findings to other contexts. However, in this study, we attempted to select participants form diverse backgrounds, including academia, executive positions, and researchers professionals. Our initial selection of indicators was conducted based on a comprehensive review of the literature and these indicators were subsequently validated based on several criteria. Nevertheless, practical and feasibility constraints related to data collection and the availability of relevant data may vary across countries. Individual nations may need to reassess key indicators according to their infrastructures like national registries, information systems and data linkage across organizations.

## Conclusion

This study identified and categorized using Donabedian model a robust set of 30 key performance indicators (KPIs) for cancer screening programs, with a high overall content validity index. Common outcome indicators across breast, cervical, and colorectal cancers included: screening coverage, invitation coverage, all-cause mortality, cause-specific mortality, invasive cancer detection rate, interval cancer rate, ductal carcinoma *in situ* (DCIS) rate, cancer detection rate, episode sensitivity, and positive predictive value for cancer detection (PPV). Although our study provides Key indicators that can applicable to evaluate the performance of cancer screening programs. The implication of our priority ranking depends on the countries’ current practice of systematic data collection and regular monitoring. We recommend that countries without systematic approach of monitoring should primarily design their system to collect at least the defined key indicators. For countries where a systematic approach for data collection is already in place, our priority ranking should be considered as a checklist, by which monitoring procedures can be verified or, if necessary, further updated.

## Data Availability

The original contributions presented in the study are included in the article/Supplementary material, further inquiries can be directed to the corresponding author.
