# Supplementary material for: Developing and validating key performance indicators for breast, cervical, and colorectal cancer screening programs: a literature review and Delphi survey
Source: Front Public Health. 2025 Apr 3;13:1491226. doi: 10.3389/fpubh.2025.1491226 (PMC12004409; doi:10.3389/fpubh.2025.1491226)
Supplement: Supplementary file 1 [file Supplementary_file_1.docx]

Appendix 1: the search strategy

| PubMed search (2023/11/22) | |
| --- | --- |
| Neoplasms OR Neoplasia OR cancer OR Tumor OR Tumors OR malignant OR carcinoma OR "Malignant Neoplasm" OR "Benign Neoplasm" OR "Inflammatory Neoplasm" OR "Malignant Tumor" | 5,724,231 |
| "Early Diagnosis" OR "early detection" OR "Screening" OR "Screening Test" OR "Diagnostic Screening" OR "Screening program" OR "screening guideline" OR " Screening Trigger" | 1,003,709 |
| ""Program Evaluat*" OR "Program Assess*" OR "Program Sustainability" OR "Program Effective*" OR "Program Appropriateness" OR "Program indicator*" OR "Program monitor*" OR "Program metric"*" | 76,027 |
| ((Neoplasms OR Neoplasia OR cancer OR Tumor OR Tumors OR malignant OR carcinoma OR "Malignant Neoplasm" OR "Benign Neoplasm" OR "Inflammatory Neoplasm" OR "Malignant Tumor") AND ("Early Diagnosis" OR "early detection" OR "Screening" OR "Screening Test" OR "Diagnostic Screening" OR "Screening program" OR "screening guideline" OR " Screening Trigger")) AND ("Program Evaluat*" OR "Program Assess*" OR "Program Sustainability" OR "Program Effective*" OR "Program Appropriateness" OR "Program indicator*" OR "Program monitor*" OR "Program metric*")) | 1,605 |

| Scoupus search (2023/11/22) | |
| --- | --- |
| TITLE-ABS-KEY ( "Neoplasms" OR "Neoplasia" OR "cancer" OR "Tumor" OR "Tumors" OR "malignant" OR "carcinoma" OR "Malignant Neoplasm" OR "Benign Neoplasm" OR "Inflammatory Neoplasm" OR "Malignant Tumor" ) AND PUBYEAR > 1999 AND PUBYEAR < 2024 | 4,571,101 |
| TITLE-ABS-KEY ( "Early Diagnosis" OR "early detection" OR "Screening" OR "Screening Test" OR "Diagnostic Screening" OR "Screening program" OR "screening guideline" OR " Screening Trigger" ) AND PUBYEAR > 1999 AND PUBYEAR < 2025 | 1,388,856 |
| TITLE-ABS-KEY ( "Program Evaluation" OR "Program Assessment" OR "Program Sustainability" OR "Program Effectiveness" OR "Program Appropriateness" OR "Program indicators" OR "Program monitoring" OR "Program metrics" ) AND PUBYEAR > 1999 AND PUBYEAR < 2024 | 75,662 |
| ( TITLE-ABS-KEY ( "Neoplasms" OR "Neoplasia" OR "cancer" OR "Tumor" OR "Tumors" OR "malignant" OR "carcinoma" OR "Malignant Neoplasm" OR "Benign Neoplasm" OR "Inflammatory Neoplasm" OR "Malignant Tumor" ) AND PUBYEAR > 1999 AND PUBYEAR < 2024 ) AND ( TITLE-ABS-KEY ( "Early Diagnosis" OR "early detection" OR "Screening" OR "Screening Test" OR "Diagnostic Screening" OR "Screening program" OR "screening guideline" OR " Screening Trigger" ) ) AND ( TITLE-ABS-KEY ( "Program Evaluation" OR "Program Assessment" OR "Program Sustainability" OR "Program Effectiveness" OR "Program Appropriateness" OR "Program indicators" OR "Program monitoring" OR "Program metrics" ) AND PUBYEAR > 1999 AND PUBYEAR < 2024 ) | 1,496 |

| Web of science search (2023/11/22) | |
| --- | --- |
| ((ALL=(Neoplasms OR Neoplasia OR cancer OR Tumor OR Tumors OR malignant OR carcinoma OR "Malignant Neoplasm" OR "Benign Neoplasm" OR "Inflammatory Neoplasm" OR "Malignant Tumor")) AND ALL=("Early Diagnosis" OR "early detection" OR "Screening" OR "Screening Test" OR "Diagnostic Screening" OR "Screening program" OR "screening guideline" OR " Screening Trigger" )) AND ALL=("Program Evaluat*" OR "Program Assess*" OR "Program Sustainability" OR "Program Effective*" OR "Program Appropriateness" OR "Program indicator*" OR "Program monitor*" OR "Program metric*") | 249 |

appendix 2: qualitative evaluation of the articles

| Quality | Score | Score based on appropriate JBI appraisal | | | | | | | | | | | Design | Author |
| --- | --- | --- | --- | --- | --- | --- | --- | --- | --- | --- | --- | --- | --- | --- |
|  |  | 11 | 10 | 9 | 8 | 7 | 6 | 5 | 4 | 3 | 2 | 1 |  |  |
| Moderate | 5 | - | - | - | Y | Y | N | Y | Y | NA | N | Y | Cross Sectional | HOFVIND, Solveig |
| High | 6 | - | - | - | Y | Y | NA | N | Y | Y | Y | Y | Cross Sectional | AKHTAR, S. S. |
| Moderate | 5 | - | - | - | Y | Y | NA | N | N | Y | Y | Y | Cross Sectional | NAUMOVIC, Tamara |
| High | 6 | - | - | - | Y | Y | NA | N | Y | Y | Y | Y | Cross Sectional | MAJEK, Ondrej |
| Moderate | 5 | - | - | - | N | Y | NA | N | Y | Y | Y | Y | Cross Sectional | ROUHOLLAHI, Mohammad Reza |
| High | 8 | - | - | - | Y | Y | Y | Y | Y | Y | Y | Y | Cross Sectional | BENTO, Maria José |
| High | 7 | - | - | - | Y | Y | Y | Y | Y | Y | N | Y | Cross Sectional | BLANKS, R. G. |
| High | 6 | - | - | - | Y | Y | NA | N | Y | Y | Y | Y | Cross Sectional | DEGROFF, Amy |
| High | 6 | - | - | - | Y | Y | NA | N | Y | Y | Y | Y | Cross Sectional | CSANÁDI, Marcell |
| High | 6 | - | - | - | Y | Y | NA | N | Y | Y | Y | Y | Cross Sectional | DAY, N. E. |
| High | 6 | - | - | - | Y | Y | NA | N | Y | Y | Y | Y | Cross Sectional | DEGROFF, Amy |
| Moderate | 7 | - | Y | N | Y | NA | N | Y | Y | Y | Y | Y | Case Control | MURILLO, Raúl |
| High | 6 | - | - | - | Y | Y | NA | N | Y | Y | Y | Y | Cross Sectional | TOMAZELLI, Jeane |
| High | 6 | - | - | - | Y | Y | NA | N | Y | Y | Y | Y | Cross Sectional | MURATOV, Sergei |
| High | 6 | - | - | - | Y | Y | NA | N | Y | Y | Y | Y | Cross Sectional | ALATAWI, Yasser M. |
| Moderate | 5 | - | - | - | Y | Y | NA | N | Y | Y | N | Y | Cross Sectional | MIRZAEI, H. |
| Moderate | 4 | - | - | - | N | Y | NA | N | Y | Y | N | Y | Cross Sectional | REBOLJ, Matejka |
| High | 6 | - | - | - | Y | Y | NA | N | Y | Y | Y | Y | Cross Sectional | BENSON, Victoria S. |
| Moderate | 8 | Y | Y | N | Y | Y | Y | N | N | Y | Y | Y | Systematic Review | LYNGE, Elsebeth |
| High | 6 | - | - | - | Y | Y | NA | N | Y | Y | Y | Y | Cross Sectional | MOTLAGH, Ali |
| High | 7 | - | - | - | Y | Y | N | Y | Y | Y | Y | Y | Cross Sectional | Zhila sadighi |
| High | 6 | - | - | - | Y | Y | NA | N | Y | Y | Y | Y | Cross Sectional | Mohammadi, G. |
| High | 5 | - | - | - | - | - | N | Y | Y | Y | Y | Y | Report | Rabeneck L |
| High | 6 | - | - | - | Y | Y | NA | N | Y | Y | Y | Y | Cross Sectional | CHOI, Eunji |
| High | 6 | - | - | - | - | - | Y | Y | Y | Y | Y | Y | Report | CANADIAN PARTNERSHIP AGAINST CANCER |
| High | 6 | - | - | - | - | - | Y | Y | Y | Y | Y | Y | Report | Australian Institute of Health and Welfare 2020 |
| High | 6 | - | - | - | - | - | Y | Y | Y | Y | Y | Y | Report | CANADA. HEALTH CANADA. |
| High | 6 | - | - | - | - | - | Y | Y | Y | Y | Y | Y | Report | Hotstone, Cathy, Stevenson |
| High | 6 | - | - | - | - | - | Y | Y | Y | Y | Y | Y | Book | SANKILA, Risto |

Appendix 3: The list of potential performance indicators

| Type | Indicator | Screening measure |
| --- | --- | --- |
| INPUT | HUMAN RESOURCE | Sufficient and available human resources |
|  |  | Number of health centers and bases where cancer screening is given |
| PROCESS | Timely diagnostic evaluation of abnormal screens | Percentage of abnormal screening results with time from screening test result to final diagnosis >60 days |
|  | Rescreening | The proportion of women aged 30–69 screened in a given year whose screening outcome was a recommendation to return for screening in 2 years and who returned for a screen within 27 months. |
|  | Recall rate | NUMERATOR: n° of women undergoing further assessment for medical reasons based on a positive screening examination (either on the same day as screening or on recall)  DENOMINATOR: n° of women screened |
|  |  | Number of smears per 1,000 women aged 20–29, per year |
|  | Public education | Number of campaigns held to inform and educate the public about cancer prevention |
|  | Participation rate | NUMERATOR: n° of women screened DENOMINATOR: n° of women invited |
|  | data availability | NUMERATOR: No. of women for whom data were available  DENOMINATOR: n° of women screened |
|  | Referral rate | NUMERATOR: No. of Women referred to GP  DENOMINATOR: n° of women screened |
|  |  | NUMERATOR: No. of Women referred to surgeon  DENOMINATOR: No. of Women referred to GP |
|  | Drop rate of people during referral from GP to surgeon | NUMERATOR: No. of Women referred to surgeon  DENOMINATOR: No. of referred from GP to surgeon |
|  | Biopsy rate | NUMERATOR: No. of biopsies  DENOMINATOR: No. of referred women to biopsy |
|  | follow-up of positive screened women | NUMERATOR: No. of followed women  DENOMINATOR: No. of women with positive/suspicious screening results |
|  | Diagnostic/therapeutic endoscopy rate | NUMERATOR: No. of with diagnostic or therapeutic endoscopy  DENOMINATOR: No. of tested population |
|  | Proportion of colonoscopies | NUMERATOR: No. of colonoscopies  DENOMINATOR: No. of people referred for colonoscopy |
|  | Mastectomy rate | NUMERATOR: n° of women with mastectomy  DENOMINATOR: n° of women screened |
|  | Tumor diameters (n) | n° of %<10 mm  n° of % < 15 mm  n° of % > 20 mm |
|  | Tumor graded (n) | n° of % grade I  n° of % grade II  n° of % grade III |
| OUTCOME | Screening coverage | NUMERATOR: n° of women screened DENOMINATOR: n° of eligible (or target) women within a given period |
|  | Invitation coverage | Total number of eligible women invited to participate in the organized screening programme |
|  | Mortality (all cause) | NUMERATOR: n° of 30-day all-cause mortality  DENOMINATOR: n° of tested population |
|  | cause-specific mortality | NUMERATOR: n° of deaths from the given cancer in a population |
|  | Invasive cancer detection rate | NUMERATOR: n° invasive screen-detected cancers  DENOMINATOR: n° of women screened |
|  | Interval cancer rate | NUMERATOR: n° of interval cancers DENOMINATOR: n° of screened negative women at the last screening round |
|  | ductal carcinoma in situ (DCIS) | NUMERATOR: n° of DCIS cancers DENOMINATOR: n° of cancer detected womens |
|  | Cancer detection rate | NUMERATOR: n° of all malignant cancers detected every 1,000 screened women  DENOMINATOR: n° of women screened |
|  | Polyp detection rate | NUMERATOR: n° of people with polyps  DENOMINATOR: n° of tested population |
|  | FOBt positivity rate | NUMERATOR: n° of people with positive FOBt  DENOMINATOR: n° of tested population |
|  | Adenoma detection rate | NUMERATOR: n° of people with adenomas  DENOMINATOR: n° of tested population |
|  | Positive predictive value for cancer detection(PPV) | NUMERATOR: the ratio of lesions that are truly positive  DENOMINATOR: those with positive test |
|  | false positive rate at screening | NUMERATOR: n° of normal cases in the surgeon's examination  DENOMINATOR: n° of women referred to a surgeon |
|  | Episode sensitivity | NUMERATOR: n° of screen-detected cancers  DENOMINATOR: n° of all cancers detected |

Appendix 4: General information of the included study

| country | frequency | Cancer type | frequency | Type of article | frequency |
| --- | --- | --- | --- | --- | --- |
| USA | 3 (10.3%) | Breast cancer | 16 (55.1%) | Descriptive (cross-sectional) | 21 (72.4%) |
| Norway | 1 (3.4%) |  |  |  |  |
| Saudi Arabia | 2 (6.9%) |  |  |  |  |
| Serbia | 1 (3.4%) |  |  | report | 5 (17.2%) |
| Czech Republic | 1 (3.4%) | Cervical cancer | 3 (10.3%) |  |  |
| Iran | 5 (17.2%) |  |  |  |  |
| Portugal | 1 (3.4%) | colorectal cancer | 2 (6.9%) | Review article | 1 (3.4%) |
| UK | 2 (6.9%) |  |  |  |  |
| Budapest | 1 (3.4%) | Breast, Cervical and colorectal cancer | 6 (20.7%) |  |  |
| Colombia | 1 (3.4%) |  |  |  |  |
| Brazil | 1 (3.4%) |  |  | Case-control article | 1 (3.4%) |
| Netherlands | 3 (10.3%) |  |  |  |  |
| Canada | 3 (10.3%) | Breast and Cervical cancer | 2 (6.9%) |  |  |
| Korea | 1 (3.4%) |  |  | Manuscript | 1 (3.4%) |
| Australia | 2 (6.9%) |  |  |  |  |
| Europe | 1 (3.4%) |  |  |  |  |

| row | Study ID | Title | Year | study-design- | country | type-cancer | tool | criteria/ indicator |
| --- | --- | --- | --- | --- | --- | --- | --- | --- |
| 1 | HOFVIND | Using the European guidelines to evaluate the Norwegian breast cancer screening program. | 2016 | Deceptive | Norway | Breast Cancer | National indicators of assessment of the Norwegian breast cancer screening program | Proportion of recalls for further examination due to mammography findings among those screened |
|  |  |  |  |  |  |  |  | proportion of biopsies among the recall examinations due to mammography findings |
|  |  |  |  |  |  |  |  | proportion of breast cancers diagnosed among the biopsies due to mammography findings |
|  |  |  |  |  |  |  |  | breast cancer detection rate per 1,000 screened due to mammography findings |
|  |  |  |  |  |  |  |  | Recalls due to mammographic findings |
|  |  |  |  |  |  |  |  | Biopsies in all recalls |
|  |  |  |  |  |  |  |  | Screen detection rate of invasive cancers/background |
|  |  |  |  |  |  |  |  | Interval cancers |
|  |  |  |  |  |  |  |  | Detection rate of invasive interval cancer/background |
| 2 | AKHTAR, S. | First organized screening mammography programme in Saudi Arabia: preliminary analysis of pilot round | 2010 | Deceptive | Saudi Arabia | Breast Cancer |  | No. of women screened |
|  |  |  |  |  |  |  |  | No. of women for whom data were available |
|  |  |  |  |  |  |  |  | No. of women recalled |
|  |  |  |  |  |  |  |  | Biopsy rate (%) |
|  |  |  |  |  |  |  |  | Biopsy rate among recalled patients (%) |
|  |  |  |  |  |  |  |  | Total no. of cancers detected |
|  |  |  |  |  |  |  |  | No. of invasive cancers |
|  |  |  |  |  |  |  |  | Cancer detection rate per 1000 screened |
|  |  |  |  |  |  |  |  | DCIS per 1000 screened |
|  |  |  |  |  |  |  |  | DCIS (% of malignant cases) |
| 3 | NAUMOVIC | Performance indicators collected from primary health centres included in organised cervical cancer screening programme in the Republic of Serbia | 2015 | Deceptive | Serbia | cervical cancer |  | Total number of eligible women invited to participate in the organized screening programme |
|  |  |  |  |  |  |  |  | Total number of eligible women screened within the screening interval |
|  |  |  |  |  |  |  |  | Total number of eligible women screened within the screening interval |
| 4 | MAJEK | Breast cancer screening in the Czech Republic: time trends in performance indicators during the first seven years of the organised programme | 2011 | Desceptive | Czech Republic | Breast cancer |  | Number of women screened |
|  |  |  |  |  |  |  |  | Breast cancer detection rate |
|  |  |  |  |  |  |  |  | Further assessment rate |
|  |  |  |  |  |  |  |  | Recall rate |
|  |  |  |  |  |  |  |  | Benign to malignant open biopsy ratio |
|  |  |  |  |  |  |  |  | Advanced cases proportion |
|  |  |  |  |  |  |  |  | Invasive cases proportion |
|  |  |  |  |  |  |  |  | Proportion among invasive |
| 5 | ROUHOLLAHI, Mohammad Reza | Situation analysis of the national comprehensive cancer control program (2013) in the ir of Iran, assessment and recommendations based on the IAEA impact mission. | 2014 | Deceptive | Iran | Breast-cervical-colon cancer | imPACT review | Surveillance systems for occupational carcinogens |
|  |  |  |  |  |  |  |  | Assessment of cancer incidence and mortality trends |
|  |  |  |  |  |  |  |  | Assessment of the stage of cancer |
|  |  |  |  |  |  |  |  | Surveillance system of the most common non-communicable risk factors |
|  |  |  |  |  |  |  |  | Clear process and outcome indicators for monitoring an evaluation |
|  |  |  |  |  |  |  |  | Resources to support cancer services at the secondary and tertiary care level |
|  |  |  |  |  |  |  |  | Basic palliative care |
|  |  |  |  |  |  |  |  | Payment Percentage as out of pocket for diagnosis and treatment |
|  |  |  |  |  |  |  |  | IT support for monitoring of cancer incidence and mortality trends |
|  |  |  |  |  |  |  |  | IT support for surveillance system of the most common NCD risk factor |
|  |  |  |  |  |  |  |  | IT support for hospital-based cancer registration |
|  |  |  |  |  |  |  |  | Oncology nurse |
|  |  |  |  |  |  |  |  | Palliative care specialists |
|  |  |  |  |  |  |  |  | Policy and managerial guidelines for cancer prevention and control public health programs |
|  |  |  |  |  |  |  |  | Clinical guidelines for pediatric cancers that are curable or treatable but not curable |
|  |  |  |  |  |  |  |  | Clinical guidelines for cancers in adults that are curable or treatable but not curable |
|  |  |  |  |  |  |  |  | Pain and palliative care clinical guidelines for children |
|  |  |  |  |  |  |  |  | Essential list of medicines for chemotherapy |
|  |  |  |  |  |  |  |  | Technology assessment for the radiotherapy devices |
| 6 | BENTO, Maria José | Performance indicators evaluation of the population-based breast cancer screening programme in Northern Portugal using the European Guidelines | 2015 | Deceptive | Portugal | breast |  | Screened women |
|  |  |  |  |  |  |  |  | Recall rate |
|  |  |  |  |  |  |  |  | Women referred to hospital |
|  |  |  |  |  |  |  |  | Screen cancers (n) |
|  |  |  |  |  |  |  |  | Screen cancers (/1000) |
|  |  |  |  |  |  |  |  | Invasive (/1000) |
|  |  |  |  |  |  |  |  | Ductal in situ (/1000) |
|  |  |  |  |  |  |  |  | Invasive (%) |
|  |  |  |  |  |  |  |  | Positive predictive value (%) |
|  |  |  |  |  |  |  |  | Ratio benign/malignant |
|  |  |  |  |  |  |  |  | Tumor diameter (n) |
|  |  |  |  |  |  |  |  | Tumor graded (n) |
| 7 | BLANKS, R. G. | Results from the UK NHS breast screening programme 1994–1999. | 2000 | randomized controlled trial | UK | breast |  | UPTAKE AND REFERRAL RATES |
|  |  |  |  |  |  |  |  | CANCER DETECTION RATES |
|  |  |  |  |  |  |  |  | NON-OPERATIVE DIAGNOSIS |
| 8 | DEGROFF, Amy | When performance management works: A study of the National Breast and Cervical Cancer Early Detection Program | 2014 | mixed-method | USA. | Breast and Cervical Cancer | Questionnaire developed by the researcher | Cervical cancer screenings provided to priority population |
|  |  |  |  |  |  |  |  | Breast cancer screenings provided to priority population |
|  |  |  |  |  |  |  |  | Complete diagnostic evaluation of abnormal cervical screenings |
|  |  |  |  |  |  |  |  | Timely diagnostic evaluation of abnormal cervical screenings |
|  |  |  |  |  |  |  |  | Treatment initiated for cervical cancers and precancerous cervical lesions |
|  |  |  |  |  |  |  |  | Timely treatment initiated for precancerous cervical lesions |
|  |  |  |  |  |  |  |  | Timely treatment initiated for invasive cervical cancers |
|  |  |  |  |  |  |  |  | Complete diagnostic evaluation of abnormal breast screens |
|  |  |  |  |  |  |  |  | Timely diagnostic evaluation of abnormal breast screens |
|  |  |  |  |  |  |  |  | Treatment initiated for breast cancers |
|  |  |  |  |  |  |  |  | Timely treatment initiated for breast cancers |
| 9 | CSANÁDI, Marcell | Key indicators of organized cancer screening programs: results from a Delphi study | 2019 | Deceptive | Budapest. | breast, cervical, or colorectal cancer | Delphi | interval cancer rate |
|  |  |  |  |  |  |  |  | detection rate |
|  |  |  |  |  |  |  |  | screening attendance |
|  |  |  |  |  |  |  |  | screening coverage |
|  |  |  |  |  |  |  |  | incidence |
|  |  |  |  |  |  |  |  | cause-specific mortality |
|  |  |  |  |  |  |  |  | The proportion of persons attending a further assessment after a positive screen test result |
|  |  |  |  |  |  |  |  | The proportion of persons attending a treatment after a diagnosis |
|  |  |  |  |  |  |  |  | invitation coverage |
|  |  |  |  |  |  |  |  | The distribution of cancers by the mode of detection |
|  |  |  |  |  |  |  |  | The proportion of complications related to the referral examinations |
|  |  |  |  |  |  |  |  | The proportion of complications related to the screening test |
| 10 | DAY, N. E. | Breast cancer screening programmes: the development of a monitoring and evaluation system | 2000 | Desceptive | Cambridge, UK. | Breast cancer |  | Compliance rate |
|  |  |  |  |  |  |  |  | Prevalence rate at initial screening test |
|  |  |  |  |  |  |  |  | Rate of interval cancers |
|  |  |  |  |  |  |  |  | Stage (or size) distribution of screen detected cancers: (1) at initial screen; (2) at subsequent screen |
|  |  |  |  |  |  |  |  | Rate of advanced cancers |
|  |  |  |  |  |  |  |  | Breast cancer death rate |
| 11 | DEGROFF, Amy | Identifying promising practices for evaluation: the National Breast and cervical Cancer early detection program | 2015 | Desceptive | USA | Breast and cervical Cancer |  | Reach to target population |
|  |  |  |  |  |  |  |  | Staff and organizational capacity |
| 12 | MURILLO, Raúl | Comprehensive evaluation of cervical cancer screening programs: the case of Colombia | 2011 | case-control study | Colombia | cervical cancer |  | screening services supply |
|  |  |  |  |  |  |  |  | screening coverage |
|  |  |  |  |  |  |  |  | conventional cytology quality |
|  |  |  |  |  |  |  |  | follow-up of positive screened women |
|  |  |  |  |  |  |  |  | Pap-smear collection centers |
|  |  |  |  |  |  |  |  | Nurses per 1000 women |
|  |  |  |  |  |  |  |  | Pathologist/cytotechnologist rate |
| 13 | TOMAZELLI, Jeane | Evaluation of breast cancer screening indicators in the female population using the National Health System, Brazil, 2018-2019: a descriptive study | 2023 | descriptive study | Brazil, | breast cancer |  | percentage distribution of screening mammogram results |
|  |  |  |  |  |  |  |  | follow-up mammogram results (BI-RADS® category) |
|  |  |  |  |  |  |  |  | breast histopathology examination results. |
|  |  |  |  |  |  |  |  | The proportion of women undergoing biopsy following screening |
|  |  |  |  |  |  |  |  | percentage of BI-RADS 0, 4 and 5 mammograms, |
|  |  |  |  |  |  |  |  | the biopsy indication rate |
|  |  |  |  |  |  |  |  | the cancer detection rate |
|  |  |  |  |  |  |  |  | the positive mammogram rate |
| 14 | MURATOV, Sergei | Monitoring and evaluation of breast cancer screening programmes: selecting candidate performance indicators | 2020 | descriptive | Netherlands | breast cancer |  | Screening coverage |
|  |  |  |  |  |  |  |  | Participation rate |
|  |  |  |  |  |  |  |  | Recall rate |
|  |  |  |  |  |  |  |  | Breast cancer detection rate (4a: initial and 4b: subsequent screenings) |
|  |  |  |  |  |  |  |  | Invasive breast cancer detection rate |
|  |  |  |  |  |  |  |  | Cancers > 20mm |
|  |  |  |  |  |  |  |  | Cancers ≤ 10mm |
|  |  |  |  |  |  |  |  | Lymph node status |
|  |  |  |  |  |  |  |  | Interval cancer rate |
|  |  |  |  |  |  |  |  | Episode sensitivity |
|  |  |  |  |  |  |  |  | Time interval between screening and first treatment |
|  |  |  |  |  |  |  |  | Benign open surgical biopsy rate |
|  |  |  |  |  |  |  |  | Mastectomy rate |
| 15 | ALATAWI, Yasser M | Evaluation of participation and performance indicators in a breast cancer screening program in Saudi Arabia | 2022 | descriptive | Saudi Arabia | breast cancer |  | Participation rate |
|  |  |  |  |  |  |  |  | Recall rate |
|  |  |  |  |  |  |  |  | Rate of diagnostic workups: Ultrasound alone |
|  |  |  |  |  |  |  |  | Rate of diagnostic workups: Ultrasound with mammography |
|  |  |  |  |  |  |  |  | Biopsy rate |
|  |  |  |  |  |  |  |  | Detection rate |
|  |  |  |  |  |  |  |  | Percent of confirmed breast cancer diagnosis |
|  |  |  |  |  |  |  |  | Episode sensitivity |
| 16 | MIRZAEI, H. | Evaluation of pilot colorectal cancer screening programs in Iran. | 2016 | descriptive | Iran | colorectal cancer |  | Percentage of people contacted |
|  |  |  |  |  |  |  |  | The percentage of people who visited in person |
|  |  |  |  |  |  |  |  | The percentage of people consulted |
|  |  |  |  |  |  |  |  | The percentage of blood samples or tissue blocks prepared or sent |
|  |  |  |  |  |  |  |  | Percentage of colonoscopy subjects |
|  |  |  |  |  |  |  |  | Colorectal cancer detection rate |
|  |  |  |  |  |  |  |  | Polyp detection rate |
|  |  |  |  |  |  |  |  | Percentage of people with sporadic type of colon and rectal cancer ، |
|  |  |  |  |  |  |  |  | Percentage of people with familial HNPCC syndrome |
|  |  |  |  |  |  |  |  | Percentage of people with familial FAP syndrome |
| 17 | REBOLJ, Matejka | Monitoring a national cancer prevention program: successful changes in cervical cancer screening in the Netherlands | 2007 | descriptive | Netherlands | cervical cancer |  | Coverage in the target age group |
|  |  |  |  |  |  |  |  | Overall compliance to follow-up |
|  |  |  |  |  |  |  |  | Proportion of women with a follow-up advice |
|  |  |  |  |  |  |  |  | Average time in follow-up before sent back to screening (women with a follow-up smear advice) |
|  |  |  |  |  |  |  |  | Number of excess smears per year per 1,000 women in the target age group |
|  |  |  |  |  |  |  |  | Number of smears per 1,000 women aged 20–29, per year |
| 18 | BENSON, Victoria S. | Toward standardizing and reporting colorectal cancer screening indicators on an international level: The International Colorectal Cancer Screening Network | 2012 | descriptive | Common between America and England | colorectal cancer | Questionnaire developed by the researcher | Coverage rate |
|  |  |  |  |  |  |  |  | Participation rate |
|  |  |  |  |  |  |  |  | FOBt inadequacy rate |
|  |  |  |  |  |  |  |  | FOBt positivity rate |
|  |  |  |  |  |  |  |  | Diagnostic/therapeutic endoscopy rate |
|  |  |  |  |  |  |  |  | Cancer detection rate (Positive predictive value for cancer detection) |
|  |  |  |  |  |  |  |  | Polyp detection rate (Positive predictive value for polyp detection) |
|  |  |  |  |  |  |  |  | Adenoma detection rate (Positive predictive value for adenoma detection) |
|  |  |  |  |  |  |  |  | Mortality (all cause) |
|  |  |  |  |  |  |  |  | Mortality (colonoscopy-specific) |
| 19 | LYNGE, Elsebeth | Reporting of performance indicators of mammography screening in Europe. | 2003 | review | Denmark, Netherlands, UK. | breast cancer |  | Coverage |
|  |  |  |  |  |  |  |  | Participation |
|  |  |  |  |  |  |  |  | Recall for assessment |
|  |  |  |  |  |  |  |  | Referral to surgery |
|  |  |  |  |  |  |  |  | Detection, invasive cancer and ductal carcinoma in situ (DCIS) |
|  |  |  |  |  |  |  |  | DCIS as the proportion of all cases detected |
|  |  |  |  |  |  |  |  | Tumor diameter, axillary lymph node and malignancy grade in screen detected invasive cancers |
|  |  |  |  |  |  |  |  | Breast conserving surgery of screen detected cases False-positive screening mammography |
|  |  |  |  |  |  |  |  | Benign surgery (surgery in women without invasive cancer or DCIS) |
|  |  |  |  |  |  |  |  | Proportionate interval cancer rate. |
| 20 | MOTLAGH, Ali | IRAN National Cancer Control Program (IrNCCP): Goals, Strategies, and Programs. | 2022 | cross sectional | Iran | Colon, breast and cervical cancer |  | Number of campaigns held to inform and educate the public about cancer prevention |
|  |  |  |  |  |  |  |  | Number of health centers and bases where public education packages are taught |
|  |  |  |  |  |  |  |  | Percentage of anti-HPV vaccination coverage relative to the total target population of the program |
|  |  |  |  |  |  |  |  | Availability of clinical practice guidelines as well as protocols for managing early detection services for Breast cancer |
|  |  |  |  |  |  |  |  | Proportion of people evaluated in level 2 to all the people in need of evaluation in the early detection program for Colorectal cancer |
|  |  |  |  |  |  |  |  | Availability of clinical practice guidelines as well as protocols for managing early detection services for Cervical cancer |
|  |  |  |  |  |  |  |  | Average time between level 1 and 2 evaluations for those in need of referral in the Breast cancer early detection program |
|  |  |  |  |  |  |  |  | Availability of clinical practice guidelines as well as protocols for managing early detection services for Colorectal cancer |
|  |  |  |  |  |  |  |  | Average time between level 1 and 2 evaluations for those in need of referral in the Cervical cancer early detection program |
|  |  |  |  |  |  |  |  | Proportion of women 30-69 years old who have been evaluated at least once in the Breast cancer early detection program in the healthcare network |
|  |  |  |  |  |  |  |  | Average time between level 1 and 2 evaluations for those in need of referral in the Colorectal cancer early detection program |
|  |  |  |  |  |  |  |  | Proportion of women 30-49 years old who have been evaluated at least once in the Cervical cancer early detection program in the healthcare network |
|  |  |  |  |  |  |  |  | Proportion of patients affected with Breast cancer identified in the framework of national Breast cancer early detection program to all newly diagnosed breast cancer cases in each year |
|  |  |  |  |  |  |  |  | Proportion of men and women 50-69 years old who have been evaluated at least once in the Breast cancer early detection program in the healthcare network |
|  |  |  |  |  |  |  |  | Proportion of patients affected with Cervical cancer identified in the framework of national Cervical cancer early detection program to all newly diagnosed Cervical cancer cases in each year |
|  |  |  |  |  |  |  |  | Proportion of people evaluated in level 2 to all the people in need of evaluation in the early detection program for Breast cancer |
|  |  |  |  |  |  |  |  | Proportion of patients affected with Colorectal cancer identified in the framework of national Colorectal cancer early detection program to all newly diagnosed Colorectal cancer cases in each year |
|  |  |  |  |  |  |  |  | Proportion of people evaluated in level 2 to all the people in need of evaluation in the early detection program for Cervical cancer |
| 21 | Zila Siddiqui | Evaluation of the integration program of breast cancer screening in the country's primary health care system | 2002 | Descriptive | Iran | breast | Checklist made by the researcher | The amount of population of women eligible for the program |
|  |  |  |  |  |  |  |  | The amount of population screened at the first level |
|  |  |  |  |  |  |  |  | Suspicious rate of cases examined at the first level |
|  |  |  |  |  |  |  |  | The drop rate of people during referral from the first level to the general practitioner (second level) |
|  |  |  |  |  |  |  |  | False positive rate (FP) in the first level |
|  |  |  |  |  |  |  |  | The predictive value of a positive outcome for first-level performance |
|  |  |  |  |  |  |  |  | Drop rate of people during referral from general practitioner to surgeon |
|  |  |  |  |  |  |  |  | The false positive rate at the second level |
|  |  |  |  |  |  |  |  | The predictive value of a positive result for the second level |
|  |  |  |  |  |  |  |  | The effectiveness of the program's referral system performance in relation to the identification of mass or lesion suspected of malignancy |
|  |  |  |  |  |  |  |  | The effectiveness of program referral system in relation to identifying malignant cases |
|  |  |  |  |  |  |  |  | The effectiveness of identifying malignant cases |
|  |  |  |  |  |  |  |  | Determination of interval cases |
|  |  |  |  |  |  |  |  | The sensitivity and specificity of the screening method |
|  |  |  |  |  |  |  |  | Detection rate of malignant cases/ and by age groups |
|  |  |  |  |  |  |  |  | Detection rate of malignant cases/ and by age groups |
|  |  |  |  |  |  |  |  | Percentage of age groups in all detected cases of breast cancer |
|  |  |  |  |  |  |  |  | The percentage of breast cancer stages in all detected cases of breast cancer and by age groups |
|  |  |  |  |  |  |  |  | The amount of lumpy cases in general physician examination |
|  |  |  |  |  |  |  |  | fna coverage rate |
|  |  |  |  |  |  |  |  | FNA referral rate (from general practitioner to surgeon) |
|  |  |  |  |  |  |  |  | Frequency distribution of fna answers |
|  |  |  |  |  |  |  |  | Biopsy referral rate by the surgeon |
|  |  |  |  |  |  |  |  | Frequency distribution of biopsy responses by FNA responses |
|  |  |  |  |  |  |  |  | Validity or sensitivity, specificity and predictive value of fna |
| 22 | Mohammadi, G. | Quality assessment of the national cancer registry in Iran: completeness and validity.‏ | 2016 | cross-sectional | Iran | Colon, breast and cervical cancer |  | Completeness of coverage |
|  |  |  |  |  |  |  |  | Death certificate only (DCO |
|  |  |  |  |  |  |  |  | Morphological verification |
|  |  |  |  |  |  |  |  | Mortality –to- incidence ratio |
|  |  |  |  |  |  |  |  | Unknown primary site |
| 23 | Rabeneck L | Assessment of a Cancer Screening Program, | 2015 | Descriptive | Canada |  |  | Participation rate |
|  |  |  |  |  |  |  |  | Follow-up colonoscopy compliance |
|  |  |  |  |  |  |  |  | Colonoscopy completion to the cecum |
|  |  |  |  |  |  |  |  | Proportion of screen-detected cancers that are Stage A |
|  |  |  |  |  |  |  |  | Endoscopic complications |
| 24 | CHOI, Eunji | Effectiveness of the Korean National Cancer Screening Program in reducing breast cancer mortality | 2021 | Prospective cohort | Korea | breast cancer |  | Incidence in Invasive breast cancer of Screened cohort |
|  |  |  |  |  |  |  |  | Incidence in Invasive breast cancer of non-screened cohort |
|  |  |  |  |  |  |  |  | Both ductal carcinoma in situ and invasive breast cancer of Screened cohort |
|  |  |  |  |  |  |  |  | Both ductal carcinoma in situ and invasive breast cancer of non-screened cohort |
|  |  |  |  |  |  |  |  | Mortality cause of Breast cancer Screened cohort |
|  |  |  |  |  |  |  |  | Mortality cause of Breast cancer non-screened cohort |
|  |  |  |  |  |  |  |  | All cause of mortality except from breast cancer Screened cohort |
|  |  |  |  |  |  |  |  | All cause of mortality except from breast cancer non-screened cohort |
| 25 | CANADIAN PARTNERSHIP AGAINST CANCER. | Breast Cancer Screening in Canada: Monitoring and Evaluation of Quality Indicators | 2017 | report | Canada | Breast Cancer |  | Number of screens |
|  |  |  |  |  |  |  |  | Number of first screens |
|  |  |  |  |  |  |  |  | Number of screen-detected cancers |
|  |  |  |  |  |  |  |  | Participation rate within a 30-month period (%) |
|  |  |  |  |  |  |  |  | Retention rate (% screened within 30 months of an initial screen) |
|  |  |  |  |  |  |  |  | Retention rate (% screened within 30 months of a subsequent screen) |
|  |  |  |  |  |  |  |  | Annual screening rate (% screened within 18 months of an initial screen) |
|  |  |  |  |  |  |  |  | Annual screening rate (% screened within 18 months of a subsequent screen) |
|  |  |  |  |  |  |  |  | Abnormal call rate (%), initial screen |
|  |  |  |  |  |  |  |  | Abnormal call rate (%), subsequent screen |
|  |  |  |  |  |  |  |  | Invasive cancer detection rate (per 1,000 screens), initial screen |
|  |  |  |  |  |  |  |  | Invasive cancer detection rate (per 1,000 screens), subsequent screen |
|  |  |  |  |  |  |  |  | In situ cancer detection, initial screen (per 1,000 screens |
|  |  |  |  |  |  |  |  | In situ cancer detection, initial screen, % in situ |
|  |  |  |  |  |  |  |  | In situ cancer detection, subsequent screen (per 1,000 screens |
|  |  |  |  |  |  |  |  | In situ cancer detection, subsequent screen, % in situ |
|  |  |  |  |  |  |  |  | Diagnostic interval (%), notified within 2 weeks of screen |
|  |  |  |  |  |  |  |  | Diagnostic interval (%), first diagnostic assessment within 3 weeks |
|  |  |  |  |  |  |  |  | Diagnostic interval (%), final diagnosis (with no tissue biopsy) within 5 weeks |
|  |  |  |  |  |  |  |  | Diagnostic interval (%), final diagnosis (with tissue biopsy) within 7 weeks |
|  |  |  |  |  |  |  |  | Positive predictive value (%), initial screen |
|  |  |  |  |  |  |  |  | Positive predictive value (%), subsequent screen |
|  |  |  |  |  |  |  |  | Non-malignant biopsy rate, initial screen (per 1,000 screens |
|  |  |  |  |  |  |  |  | Non-malignant biopsy rate, initial screen, % open |
|  |  |  |  |  |  |  |  | Non-malignant biopsy rate, subsequent screen (per 1,000 screens) |
|  |  |  |  |  |  |  |  | Non-malignant biopsy rate, subsequent screen, % open |
|  |  |  |  |  |  |  |  | Screen-detected invasive cancer tumour size (%), <=15 mm |
|  |  |  |  |  |  |  |  | Percentage of node negative screen-detected invasive cancer (%) |
|  |  |  |  |  |  |  |  | Post-screen invasive cancer rate (per 10,000 person-years), 0 to <12 months |
|  |  |  |  |  |  |  |  | Post-screen invasive cancer rate (per 10,000 person-years), 12 to 24 months |
|  |  |  |  |  |  |  |  | Sensitivity of the screening mammography program, subsequent screen |
| 26 | Australian Institute of Health and Welfare 2020. | BreastScreen Australia monitoring report 2020 | 2020 | report | Australia | Breast Cancer |  | Participation |
|  |  |  |  |  |  |  |  | Rescreening |
|  |  |  |  |  |  |  |  | Recall to assessment |
|  |  |  |  |  |  |  |  | Invasive breast cancer detection |
|  |  |  |  |  |  |  |  | Ductal carcinoma in situ (DCIS) detection |
|  |  |  |  |  |  |  |  | Interval cancers |
|  |  |  |  |  |  |  |  | Program sensitivity |
|  |  |  |  |  |  |  |  | Invasive breast cancer incidence |
|  |  |  |  |  |  |  |  | Ductal carcinoma in situ (DCIS) incidence |
|  |  |  |  |  |  |  |  | Mortality from breast cancer |
| 27 | CANADA. HEALTH CANADA | EVALUATION INDICATORS WORKING GROUP. | 2007 | Report | Canada | breast |  | PARTICIPATION RATE |
|  |  |  |  |  |  |  |  | RETENTION RATE |
|  |  |  |  |  |  |  |  | ANNUAL SCREENING RATE |
|  |  |  |  |  |  |  |  | ABNORMAL CALL RATE |
|  |  |  |  |  |  |  |  | INVASIVE CANCER DETECTION RATE |
|  |  |  |  |  |  |  |  | IN SITU CANCER DETECTION |
|  |  |  |  |  |  |  |  | DIAGNOSTIC INTERVAL |
|  |  |  |  |  |  |  |  | POSITIVE PREDICTIVE VALUE OF THE SCREENING MAMMOGRAPHY PROGRAM |
|  |  |  |  |  |  |  |  | NON-MALIGNANT BIOPSY RATE |
|  |  |  |  |  |  |  |  | SCREEN-DETECTED INVASIVE TUMOUR SIZE |
|  |  |  |  |  |  |  |  | PROPORTION OF NODE NEGATIVE SCREEN-DETECTED INVASIVE CANCER |
|  |  |  |  |  |  |  |  | POST-SCREEN INVASIVE CANCER RATE |
|  |  |  |  |  |  |  |  | SENSITIVITY OF THE SCREENING MAMMOGRAPHY PROGRAM |
| 28 | Hotstone, Cathy | BreastScreen Australia monitoring report 2000–2001 | 2003 | report | Australia | breast |  | Participation |
|  |  |  |  |  |  |  |  | Detection of small invasive cancers |
|  |  |  |  |  |  |  |  | Sensitivity |
|  |  |  |  |  |  |  |  | Ductal carcinoma in situ |
|  |  |  |  |  |  |  |  | Rescreening |
|  |  |  |  |  |  |  |  | Incidence |
|  |  |  |  |  |  |  |  | Mortality |
| 29 | SANKILA, Risto | Evaluation and monitoring of screening programmes. | 2001 | book | Europe | Breast, cervical and cervical cancer |  | attendance rate |
|  |  |  |  |  |  |  |  | recall rate |
|  |  |  |  |  |  |  |  | false positive rate at screening |
|  |  |  |  |  |  |  |  | screening sensitivity |
|  |  |  |  |  |  |  |  | screening specificity |
|  |  |  |  |  |  |  |  | biopsy rate |
|  |  |  |  |  |  |  |  | positive predictive value of biopsy |
|  |  |  |  |  |  |  |  | cancer detection rate |
|  |  |  |  |  |  |  |  | screening prevalence/expected incidence ratio |
|  |  |  |  |  |  |  |  | proportion of in situ cancers |
|  |  |  |  |  |  |  |  | proportion of invasive cancers <10 mm |
|  |  |  |  |  |  |  |  | proportion of invasive cancers >20 mm |
|  |  |  |  |  |  |  |  | proportion of cancers with positive nodal status |
|  |  |  |  |  |  |  |  | proportion of advanced cancers (Stage II or more) |
|  |  |  |  |  |  |  |  | interval cancer rate |
|  |  |  |  |  |  |  |  | absolute rate of advanced cancer detection |
|  |  |  |  |  |  |  |  | absolute rate of small cancer detection |
|  |  |  |  |  |  |  |  | evolution of prognostic factors in target population over time |

Appendix 5: Table of KPIs changes

| dimension | accepted | referred to the expert panel | Changes |
| --- | --- | --- | --- |
| input | 1 | 1 | "The number of health centers and bases providing cancer screening services was changed to the percentage of health centers and bases providing cancer screening services." |
| process | 12 | 5 | “Recall rate merged with the follow-up of women with positive screening results.” |
|  |  |  | “The number of smears per 1,000 women aged 20–29 per year was changed to the percentage of smears per 1,000 women aged 20–29 per year” |
|  |  |  | "Public education: The number of campaigns held to inform and educate the public about cancer prevention was changed to the percentage of campaigns held to inform and educate the public about cancer prevention." |
|  |  |  | “Participation rate” merged with “Screening coverage” |
|  |  |  | Mastectomy rate divided into two categories: Partial and total Mastectomy |
| outcome | 9 | 3 | Invitation coverage merged with Participation rate |
|  |  |  | "Mortality divided into two categories: all-cause mortality and cause-specific mortality." |
|  |  |  | Positive predictive value for cancer detection (PPV) merged with the false positive rate in screening |
